# Content analysis of fairness product posts on Instagram

**DOI:** 10.1111/srt.13728

**Published:** 2024-05-08

**Authors:** Shazli Razi, Isabella J. Tan, Babar Rao

**Affiliations:** ^1^ Department of Internal Medicine Jersey Shore University Medical Center Neptune New Jersey USA; ^2^ Rao Dermatology Atlantic Highlands New Jersey USA; ^3^ Center for Dermatology Rutgers Robert Wood Johnson Medical School Somerset New Jersey USA; ^4^ Department of Dermatology Weill Cornell Medicine New York New York USA

In recent years, social media platforms have become a popular medium for promoting beauty products, especially fairness products.^1^, ^2^ Fairness products refer to cosmetic products that are designed and marketed to lighten or brighten the skin tone.^1^ They play a significant role in the personal care industry, catering to individuals who desire an even complexion or specific aesthetic appearance.^1^ This research aims to analyze the promotion strategies employed by various fairness cream brands in different countries, with a specific focus on the use of hashtags, likes, and gender targeting. By investigating these factors, insights into fairness product marketing techniques used by beauty brands and their impact on consumers will be evaluated.

Data for this research was collected from Instagram, by searching for relevant posts associated with fairness products. The data includes information on the country, brand, promotion status, specific body areas targeted, hashtags used, upload date, number of likes, gender targeting, and the usage of words associated with fairness, whiteness, brightness, lightening, glow, as well as specific ingredients. A total of 124 posts were analyzed (Table 1).

**TABLE 1:** Post characteristics for fairness products on Instagram.

Post characteristics	Count
Total posts analyzed	124
Earliest upload date	2009
Posts (2020–2023)	103
**Country of origin**	
India	55
Korea	22
**Usage of terms**	
“Fairness”	6
“Bright”	30
“Glow”	43
**Presenter/speaker**	
Influencers	40
Celebrities	7
Healthcare professionals	1

Regarding the post characteristics of the promoted fairness products, the earliest upload date was 2009, with a majority of the posts (103) originating between 2020 and 2023. There were a total of 123 unique brands originating from a variety of countries, with a predominance being from India and Korea. 85% of posts specifically targeted facial fairness, 9% targeted total body, and 6% targeted other body regions, which included intertriginous regions. Six posts explicitly mentioned the term “fairness” with varying degrees, either in an oral or visual format. The terms “bright” and “glow” were used more frequently, with 30 and 43 posts directly utilizing these terms in an oral or visual format, respectively. The mention of ingredients varied across brands, with some posts highlighting specific ingredients such as niacinamide, vitamin C, and kojic acid. These posts mainly targeted female audiences (*n* = 68) (55%) (Figure 1), and often included celebrities and influencers, reflecting 7 and 40 posts, respectively. A minority of posts featured physicians or other healthcare professionals (*n* = 1) (0.8%).

**FIGURE 1:**
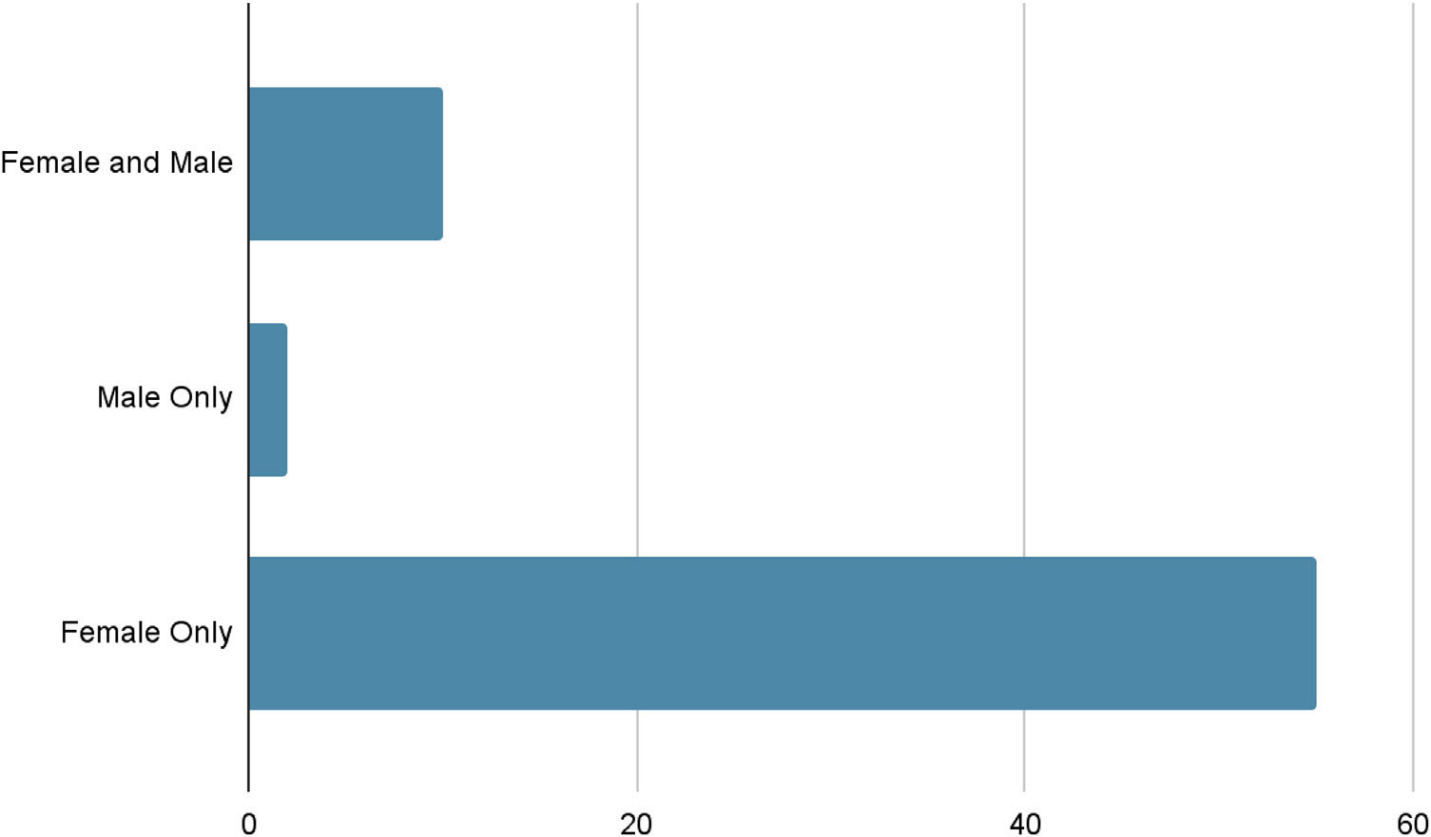
Percentage of posts targeting female and male audiences.

The findings indicate that beauty brands employ diverse promotion strategies on social media platforms, including Instagram, to engage with their target audience. The use of hashtags and keywords allows brands to reach a wider audience and increase visibility and the presence of influencers and celebrities may enhance the credibility and desirability of the products.

The analysis of the social media promotion strategies of fairness products reveals diverse approaches to marketing these products. The shift in language from explicit fairness claims to more positive terms like “bright” and “glow” indicates evolving marketing strategies and consumer perspectives. Further, the presence of celebrities and influencers in posts suggests their prominent role in brand and product promotion.

These findings contribute to the understanding of the promotional and marketing landscape surrounding fairness products and sheds light on the dynamics of consumer engagement and preferences in this domain. The study highlights the potential impact of these techniques on consumers and raises important questions pertaining to beauty standards and the marketing practices of beauty brands. Furthermore, the involvement of dermatologists in this research is crucial as they play a significant role in guiding consumers on skincare and beauty products. Dermatologists can provide expert advice and insights into the safety, efficacy, and potential side effects of fairness products. Their expertise is essential in promoting consumer awareness and education regarding these products. Further research is needed to explore the perceptions and attitudes of consumers regarding these promotion strategies and their effects on self‐image and well‐being, with particular attention to the guidance and recommendations provided by dermatologists.

## AUTHOR CONTRIBUTION

All authors have made substantial contributions. Writing: Shazli Razi and Isabella J. Tan; Review and Editing: Shazli Razi and Isabella J. Tan; Supervision: Babar Rao

## CONFLICT OF INTEREST STATEMENT

None declared.

## Data Availability

The data that support the findings of this study are available from the corresponding author upon reasonable request.
